# Mutations in the Gene Encoding the Ancillary Pilin Subunit of the *Streptococcus suis srtF* Cluster Result in Pili Formed by the Major Subunit Only

**DOI:** 10.1371/journal.pone.0008426

**Published:** 2010-01-05

**Authors:** Nahuel Fittipaldi, Daisuke Takamatsu, María de la Cruz Domínguez-Punaro, Marie-Pier Lecours, Diane Montpetit, Makoto Osaki, Tsutomu Sekizaki, Marcelo Gottschalk

**Affiliations:** 1 Groupe de Recherche sur les Maladies Infectieuses du Porc and Centre de Recherche en Infectiologie Porcine, Faculté de Médecine Vétérinaire, Université de Montréal, St-Hyacinthe, Canada; 2 Research Team for Bacterial/Parasitic Diseases, National Institute of Animal Health, National Agriculture and Food Research Organization, Tsukuba, Japan; 3 Centre de Recherche et de Développement sur les Aliments, Agriculture et Agroalimentaire Canada, St-Hyacinthe, Canada; 4 Research Center for Food Safety, Graduate School of Agricultural and Life Sciences, The University of Tokyo, Tokyo, Japan; National Institute of Allergy and Infectious Diseases, National Institutes of Health, United States of America

## Abstract

Pili have been shown to contribute to the virulence of different Gram-positive pathogenic species. Among other critical steps of bacterial pathogenesis, these structures participate in adherence to host cells, colonization and systemic virulence. Recently, the presence of at least four discrete gene clusters encoding putative pili has been revealed in the major swine pathogen and emerging zoonotic agent *Streptococcus suis*. However, pili production by this species has not yet been demonstrated. In this study, we investigated the functionality of one of these pili clusters, known as the *srtF* pilus cluster, by the construction of mutant strains for each of the four genes of the cluster as well as by the generation of antibodies against the putative pilin subunits. Results revealed that the *S. suis* serotype 2 strain P1/7, as well as several other highly virulent invasive *S. suis* serotype 2 isolates express pili from this cluster. However, in most cases tested, and as a result of nonsense mutations at the 5′ end of the gene encoding the minor pilin subunit (a putative adhesin), pili were formed by the major pilin subunit only. We then evaluated the role these pili play in *S. suis* virulence. Abolishment of the expression of *srtF* cluster-encoded pili did not result in impaired interactions of *S. suis* with porcine brain microvascular endothelial cells. Furthermore, non-piliated mutants were as virulent as the wild type strain when evaluated in a murine model of *S. suis* sepsis. Our results show that *srtF* cluster-encoded, *S. suis* pili are atypical compared to other Gram-positive pili. In addition, since the highly virulent strains under investigation are unlikely to produce other pili, our results suggest that pili might be dispensable for critical steps of the *S. suis* pathogenesis of infection.

## Introduction

*Streptococcus suis* is a major swine pathogen responsible for severe economic losses to the porcine industry [1]. This bacterium is also a zoonotic agent affecting, for the most part, people in close contact with swine or pork by-products [2]. In recent times, however, *S. suis* has strongly emerged as an important public health issue in South East and East Asia. For instance, it has been shown that this pathogen is the primary cause of adult meningitis in Vietnam [3] and the second in Thailand [4]. Moreover, in 2005, more than 200 human *S. suis* cases with a death toll of 39 were reported during a single outbreak in China [5]. In both swine and humans the main clinical manifestations of *S. suis* are meningitis and septicemia [1], [2]. Most cases of *S. suis* disease are caused by strains belonging to the serotype 2 and, therefore, almost all studies on virulence factors and pathogenesis of the infection have been carried out with this serotype [1]. It has been shown that the polysaccharide capsule is essential for the virulence of *S. suis* by allowing the bacterium to escape phagocyte killing [6]. Modifications of cell wall components such as the N-deacetylation of the peptidoglycan and the D-alanylation of lipoteichoic acids have recently been shown to contribute to the virulence of *S. suis* [7], [8]. As well, an isogenic mutant for a serum opacity-like factor has been found to be attenuated in pigs [9]. In contrast, other factors, such as a hemolysin (suilysin), the so-called extracellular protein factor (EF) and a muramidase-released protein (MRP), have been shown to be linked to, but not essential for, the virulence of *S. suis* [1].

*S. suis* needs to invade the central nervous system (CNS) in order to cause meningitis in swine. It has been proposed that, among other routes, this pathogen might reach the CNS by crossing the porcine blood-cerebrospinal fluid barrier as well as the blood-brain barrier by transcytosis through porcine choroid plexus epithelial cells and brain microvascular endothelial cells (BMEC), respectively [10], [11], [12]. In previous work using an in vitro model of *S. suis*-porcine BMEC interactions and the selective capture of transcribed sequences (SCOTS), several genes were identified which were highly upregulated by this bacterium upon contact with these host cells [13]. One of these genes was SSU_0424 encoding a putative signal peptidase [13] (the nomenclature used is that of the Sanger Institute for the very recently finished sequencing project of strain P1/7 [14]). Further *in silico* analysis showed that the three genes downstream the signal peptidase identified by SCOTS, namely SSU_0426, SSU_0427 and SSU_0428, putatively encode two cell wall sorting signal (CWSS)-containing proteins and a class C sortase, respectively [13], [15]. This genetic organization is similar to that of some described Gram positive pilus cluster [16], [17]. In a recent study, the sortase gene was renamed as *srtF* and the signal peptidase gene as *sipF* (for signal peptidase gene in the *srtF* cluster) [18]. The genes encoding the two CWSS-containing proteins were renamed as *sfp2 and sfp1* (for *srtF*-associated pilin subunit), respectively [18]; *sfp2* has been suggested to encode the putative pilin ancillary subunit (a putative adhesin) and *sfp1* the putative main pilin subunit forming the pilus backbone [13]. The full cluster was named *srtF* pilus cluster [18] and found to be highly homologous to the *Streptococcus agalactiae* (Group B *Streptococcus*, GBS) pilus island 2b [13].

Gram-positive pili participate in biofilm formation [19], [20], [21] and have been shown to play important roles in other aspects of the virulence of several invasive human streptococcal pathogens, including GBS, *Streptococcus pneumoniae* and *Streptococcus pyogenes* (Group A *Streptococcus*, GAS) [17]. For instance, at least two pneumococcal pili are involved in adherence to epithelial cells and contribute to the virulence of these organisms [22], [23], [24]. In GBS and GAS, besides contributing to phagocyte resistance and systemic survival [25], pili participate in adhesion to extracellular matrix proteins [26] and to human epithelial cells [27]. Furthermore, pili have been shown to be important for GBS adherence to and invasion of human BMEC [28]. We therefore speculated that putative pili encoded by the *srtF* cluster might play a role during the interactions of *S. suis* with porcine BMEC and, also, that they might contribute to the virulence traits of this pathogen. In this work, we characterized the *srtF* pilus cluster in a highly virulent field strain of *S. suis* serotype 2 and investigated the role that pili encoded by this cluster play in some aspects of the pathogenesis of the *S. suis* infection.

## Results

### The *srtF* Pilus Cluster Encodes Pili Formed by the Major Pilin Subunit Only

**Figure 1:**
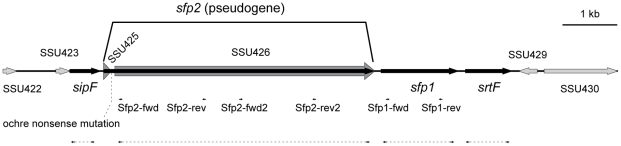
Genetic organization of the *S. suis* strain P1/7 *srtF* pilus cluster. (Available at the Sanger Institute, http://www.sanger.ac.uk/Projects/S_suis/). The *srtF* pilus cluster comprises 4 genes: *sipF* encoding a signal peptidase; *sfp2*, which is now considered to be a pseudogene formed by the previously reported coding sequences SSU_0425 and SSU_0426 (indicated by the thick dark grey arrows); *sfp1*, encoding the major pilin subunit; and *srtF*, encoding a dedicated (pilin polymerase) sortase. Small arrows indicate the annealing positions of the primers used to generate the 6xHis recombinant proteins rSfp1, rSfp2A and rSfp2B. The dashed lines surrounded by arrows indicate the regions deleted in the Δ*sipF*, Δ*sfp2*, Δ*sfp1* and Δ*srtF* mutants.

**Figure 2:**
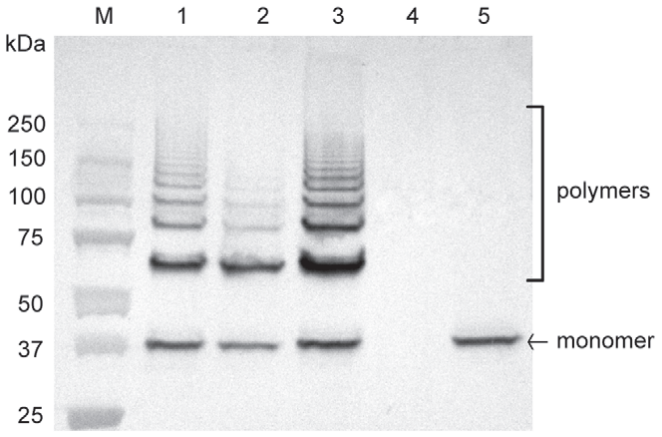
Western-blotting analysis of cell wall-anchored proteins of *S. suis* strain P1/7 and derived mutants with anti-Sfp1 antisera. Mutanolysin-extracted surface proteins were resolved on Tris-HCl Ready gradient 4–15% SDS-PAGE gels (BioRad) and transferred onto nitrocellulose membranes. Sfp1 was detected using specific rabbit antisera and horseradish peroxidase-coupled, goat anti-rabbit secondary antibodies (Jackson ImmunoResearch, West Grove, PA). Five µg of protein were loaded per well. M: Molecular weight markers. Lane 1: WT strain P1/7. Lane 2: Δ*sipF* mutant. Lane 3: Δ*sfp2* mutant. Lane 4: Δ*sfp1* mutant. Lane 5: Δ*srtF* mutant. See the results section for more details.

The genome of the virulent field strain P1/7 contains a genetic region designated as the *srtF* pilus cluster where four genes encoding a putative signal peptidase (*sipF*), putative ancillary and major pilin subunits (*sfp2* and *sfp1*, respectively) and a putative dedicated class C sortase (*srtF*) are found [13], [14], [18] (Figure 1). To assess whether the *srtF* cluster mediates formation of pili in *S. suis*, we inactivated each of the four genes by precise, in frame, allelic replacement. The resulting mutant strains exhibited growth kinetics equivalent to those of the WT parent strain upon cultivation in standard laboratory media and other media used in our in vitro assays (data not shown). At first, pili production by the WT and mutant strains was studied by Western-blotting of cell wall protein extracts (mutanolysin digests) using a specific antiserum directed against the putative pilin subunit Sfp1. In the WT sample, this antiserum recognized a band of approximately 37 kDa that likely corresponds to a Sfp1 monomer whose N-terminal signal peptide and C-terminal residues after the CWSS motif have been removed (predicted MW of 51 kDa for a native unprocessed monomer). As well, high molecular weight species that likely represent Sfp1-containing polymers were detected in the WT sample (Figure 2, lane 1). The monomer and polymers were also detected in the Δ*sipF* and Δ*sfp2* mutants (lanes 2 and 3), although the intensity of the higher molecular weight polymers was lower in the former mutant. By contrast, neither monomer nor polymers were detected in the Δ*sfp1* mutant (lane 4). On the other hand, the Sfp1 monomer, but not polymerized structures were detected in the cell wall extracts of the Δ*srtF* mutant (lane 5), indicating that polymerization of pili requires the action of the dedicated class C sortase SrtF.

**Figure 3:**
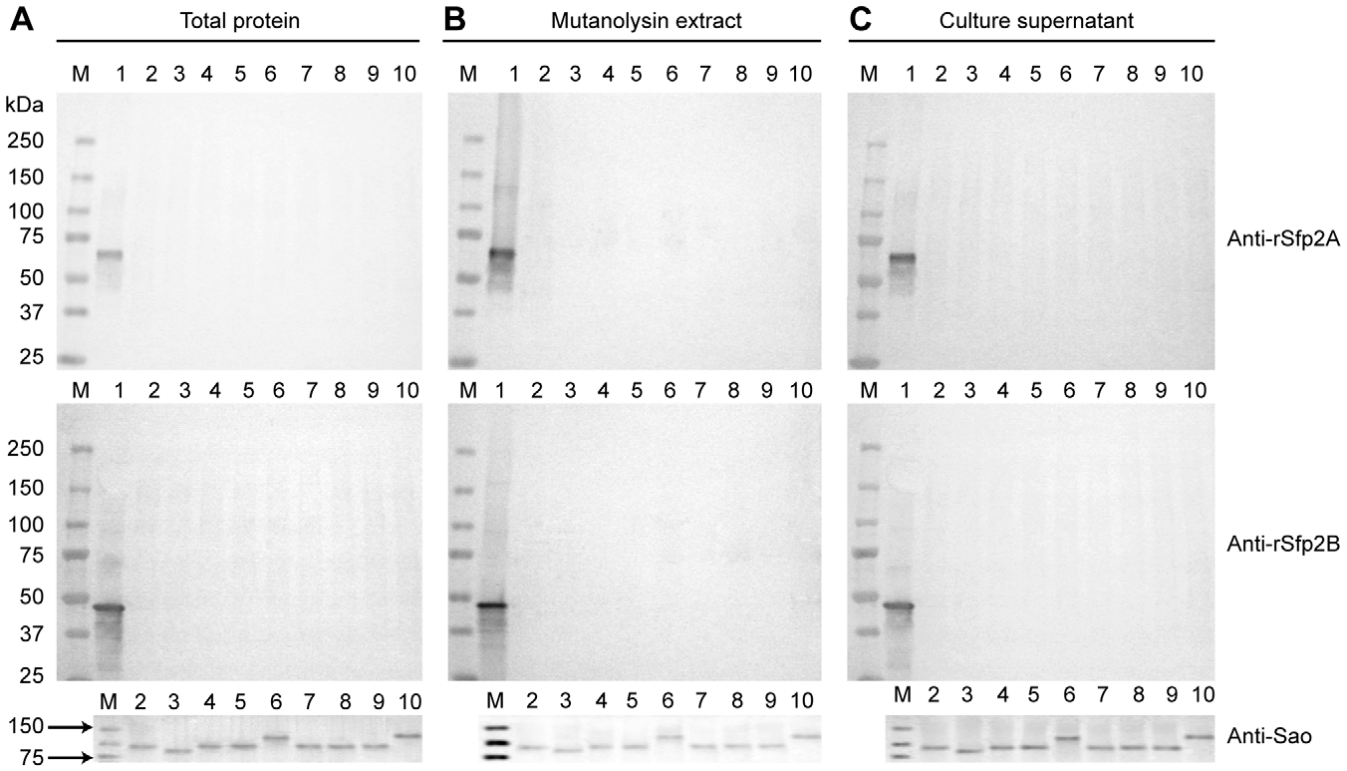
Sfp2 is not produced by *S. suis* strain P1/7 and other highly invasive serotype *S. suis* 2 isolates. Western-blotting analysis of A) whole cell protein preparations, B) mutanolysin-extracted surface protein preparations and C), 10-fold concentrated culture supernatant preparations of *S. suis* strain P1/7 and other highly invasive serotype 2 isolates with two different rabbit antisera recognizing two different recombinant 6xHis-tagged fragments of Sfp2 (rSfp2A: upper panel; rSfp2B, middle panel). Proteins were resolved on 7.5% SDS-PAGE gels and transferred onto nitrocellulose membranes. Horseradish peroxidase-coupled, goat anti-rabbit IgG (Jackson ImmunoResearch, West Grove, PA) were used as secondary antibodies. Although the recombinant 6xHis-Sfp2 fragments were recognized by the respective antisera (lanes 1), no signal corresponding to the native protein was detected in any of the tested strains. To assess the quality of the protein preparations used, detection of the known LPXTG-containing protein Sao [53], [67] was carried with an anti-Sao pAb. This protein, for which MW variants have been described, was detected in all the tested strains and fractions (lower panel). The same amount of total protein was loaded per well for each fraction. M: Molecular weight markers. Lane 1: recombinant 6xHis-Sfp2 fragments. Lane 2: Strain P1/7. Lane 3: Strain 31533. Lane 4: Strain 166. Lane 5: Strain D24. Lane 6: Strain S735 (serotype 2 reference strain). Lane 7: Strain D282. Lane 8: Strain LEF95. Lane 9: Strain HUD Limoges. Lane 10: strain 89–1591.

We then performed Western-blotting using two different antisera raised against two different fragments of Sfp2 (named rSfp2 A and B, respectively). Sfp2 is, based on homology to GBS PI-2A (37% of positives, 29% of identity to SAN_1519 of GBS COH1), the putative minor pilin subunit of the *srtF* cluster [13], [18]. Despite the fact that both recombinant proteins were recognized by the respective antisera (Figure 3A, B and C, lanes 1), we failed to detect any reactive protein in both whole cell and cell wall protein preparations, as well as in concentrated culture supernatant preparations of the WT (Figure 3A, B and C, lanes 2) and mutant strains (data not shown). Although these results were at first surprising, while this manuscript was in preparation the genome of strain P1/7 was published [16]. In the released data the gene reported here as *sfp2* (SSU_0426) and a short open reading frame (SSU_0425) upstream of *sfp2* are now annotated as a single pseudogene SSU_0425 [14], [18]. In this pseudogene a premature ochre nonsense mutation is found after codon 49 (Figure 1). Therefore, failure of our two anti-Sfp2 antisera to recognize the native Sfp2 protein confirms this new released sequence data for strain P 1/7.

### Electron Microscopy Evidence for Pilus-Like Structures

**Figure 4:**
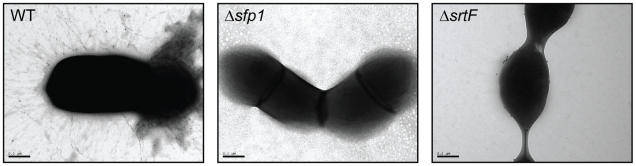
Immunogold labeling and transmission electron microscopy of pilus-like structures on the cell surface of *S. suis* P1/7 and its derived mutants. In the WT strain P1/7 (left) immunogold labeling showed the presence of thin pilus-like structures that were entirely decorated by the anti Sfp1-specific antiserum and 10 nm colloidal gold-conjugated anti-rabbit IgG antibodies. In contrast, no labeling was detected in the Δ*sfp1* mutant (center) while the Δ*srtF* mutant (right) presented labeling of the bacterial surface but not pilus-like appendages. Magnification: 60,000×.

Antisera raised against recombinant pilin proteins were also used to investigate the protein localization in the cell surface of strain P 1/7 and selected mutants by immunogold electron microscopy (IEM). As expected from the previous Western-blotting results, labeling was not observed with antibodies directed against Sfp2 (data not shown). On the other hand, confirming Western-blotting results, immunogold labeling with the antiserum specific for Sfp1 revealed long and abundant pilus-like structures extending up to 800 nm from the bacterial surface in the WT strain (Figure 4, left panel). Suggesting that Sfp1 monomers form the backbone of the pilus, pili were entirely decorated by the anti Sfp1-specific antiserum and 10 nm colloidal gold-conjugated anti-rabbit IgG antibodies. As expected, no labeling was observed for the Δ*sfp1* mutant (Figure 4, center panel), while labeling of the bacterial surface but not pilus-like structures was observed for the Δ*srtF* mutant (Figure 4, right panel). No immunogold labeling was observed using control rabbit normal antisera (data not shown).

### Evaluation of the Role of the Housekeeping Sortase SrtA in Pili Production

**Figure 5:**
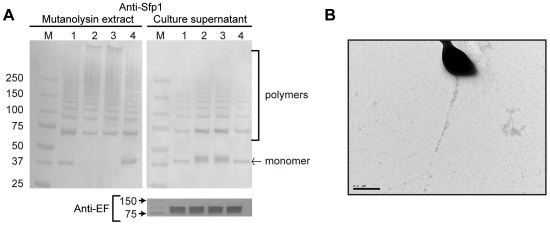
Role of *srtA* in formation of the pilus encoded by the *srtF* cluster. A) Western-blotting analysis using anti-Sfp1 antisera of cell wall-anchored proteins (left) and concentrated culture supernatants (right) of *S. suis* strain P1/7 (lane 1), its derived Δ*srtA* mutant (lane 2), a mock-complemented Δ*srtA* mutant (lane 3) and the Δ*srtA* mutant complemented *in trans* with *srtA* (lane 4) (upper panel). SDS-PAGE and Western-blotting were performed as described in Figure 2 legend. While Sfp1 polymers were detected in the cell wall fraction of all four tested strains, Sfp1 monomers were absent from the Δ*srtA* mutant and the mock-complemented Δ*srtA*. Reintroduction of the *srtA* gene into the Δ*srtA* mutant complemented the defect (left upper panel). More Sfp1 monomers and slightly more Sfp1 polymers were detected in the culture supernatant of the Δ*srtA* mutant, while the WT phenotype was restored by complementation with the *srtA* gene (right upper panel). To ensure that equal amounts of protein were loaded per well in the supernatant fraction, detection of the previously reported [68] secreted protein extracellular factor (EF) was carried out using the same samples and a previously described monoclonal antibody [55] (lower panel). M: Molecular weight markers. B) Immunogold labeling and transmission electron microscopy (magnification: 20,000×) showing scarce but very long pilus-like structures on the cell surface of the Δ*srtA* mutant. Note the differences in magnification with the microphotographs shown in Figure 4.

The housekeeping sortase SrtA has been shown to mediate anchoring of LPXTG proteins to the *S. suis* cell wall peptidoglycan [29]. We hypothesized that if SrtA was required for the attachment of pili into the cell wall peptidoglycan, then pili produced by a Δ*srtA* mutant should be released into the media in higher amounts than those produced by the WT strain. To investigate the validity of this hypothesis, we constructed a Δ*srtA* mutant by allelic exchange. When we tested production of Sfp1 polymers in the Δ*srtA* mutant by Western-blotting of cell wall proteins, the amount of Sfp1 polymers detected by our antiserum in the Δ*srtA* mutant was similar to that found in the WT strain. This result is in agreement with a previous report describing the contribution of SrtA to pili production in GBS [29] and conceivably explained by the fact that the pili subunits are assembled by the pilin polymerase SrtF irrespective of the presence of SrtA, remaining transiently associated to the cell wall. However, in contrast to the WT strain, no Sfp1 monomers could be detected in the cell wall fraction of the Δ*srtA* mutant (Figure 5A, lanes 1 and 2). The WT phenotype was restored by complementation in trans of the Δ*srtA* mutant (Figure 5A, lane 4). As expected, more Sfp1 monomers were detected in the culture supernatant of the Δ*srtA* mutant in comparison to the WT strain or the complemented Δ*srtA* mutant. In addition, the amount of Sfp1 polymers released in the culture supernatant fraction by the Δ*srtA* mutant was slightly higher than that of the WT strain or the complemented mutant (Figure 5A). We then analyzed pili production by the Δ*srtA* mutant using IEM. Figure 5B shows a representative Δ*srtA* mutant bacterial cell presenting pilus-like structures much longer than those found in the WT strain, a result that suggests that SrtA might be involved in termination of pilus assembly. Interestingly, and in contrast to the WT strain, pili produced by the Δ*srtA* mutant were scarce in the bacterial surface. Indeed, most cells presented one of these structures and many of them were devoid of pili. Together with the Western-blotting experiments, these IEM results demonstrate that the housekeeping sortase SrtA is not necessary for pilus polymerization but strongly suggest that it is required for anchoring the pilus to the cell wall.

### Production of Pili by the *srtF* Cluster in Other Virulent *S. suis* Strains

**Figure 6:**
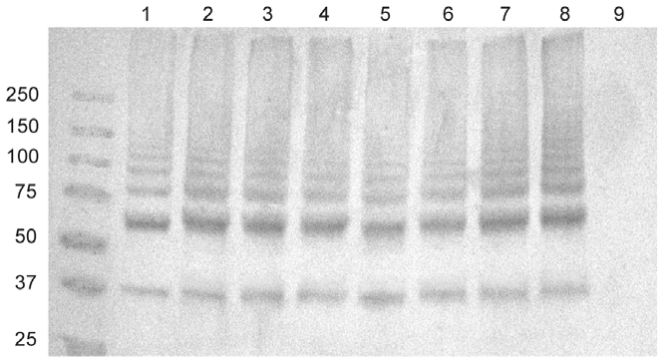
Western-blotting analysis of cell wall-anchored proteins of *S. suis* strain P1/7 and other highly invasive serotype 2 isolates with anti-Sfp1 antisera. SDS-PAGE of mutanolysin extracted surface proteins and Western-blotting were performed as described in Figure 2 legend. Five µg of protein were loaded per well. M: MW markers. Lane 1: Strain P1/7. Lane 2: Strain 31533. Lane 3: Strain 166. Lane 4: Strain D24. Lane 5: Strain S735 (serotype 2 reference strain). Lane 6: Strain D282. Lane 7: Strain LEF95. Lane 8: Strain HUD Limoges. Lane 9: Strain 89–1591. See the results section for more details.

Absence of the minor subunit in polymerized pili of strain P1/7 seems to be the consequence of a genetic conversion which resulted in inactivation of *sfp2* in this strain. Other strains might, however, present an intact *sfp2* gene. Indeed, by the use of PCR amplification with specific primers a previous study demonstrated the presence of the *srtF* pilus cluster genes in several isolates of *S. suis* serotype 2 from various sources [18]. We therefore analyzed whether the *S. suis* serotype 2 reference strain and other well-characterized highly virulent field strains (Table 1) can express pili that are formed by both Sfp1 and Sfp2 subunits. Western-blotting results showed that most of the investigated strains produced pili formed by the major pilin subunit Sfp1 only and were devoid of Sfp2 (Figures 3A, B and C, lanes 3 to 10 and Figure 6). The exception was strain 89–1591, for which we failed to detect not only Sfp2 but also Sfp1 monomers (Figure 6, lane 9), despite the fact that it has been reported that this strain contains all four *srtF* cluster genes [18]. However, further analysis of sequence data for this particular strain (available at http://genome.jgi-psf.org/draft_microbes/strsu/strsu.home.html) showed that these four genes are not organized in a typical pilus cluster but, instead, they are found at different locations in the genome (data not shown). Consistently, we failed to amplify the *srtF* pilus cluster in this strain using the primer pair PSF-ID1 and srtF-ID8 (Table S1), which anneal to the region upstream of *sipF* and downstream of *srtF*, respectively, while a fragment of approximately 9.4 kb was amplified from P1/7 and the remaining strains (Fig. S1).

**Table 1:** Bacterial strains and plasmids used in this study.

Strain/Plasmid	General characteristics	Source/Reference
*E. coli*		
TOP10	F- *mcr*A Δ(*mrr-hsd*RMS-*mcr*BC) φ80*lac*ZΔM15 Δ*lac*X74 *rec*A1 *ara*D139 Δ(*ara-leu*) 7697 *gal*U *gal*K *rps*L (StrR) *end*A1 *nup*G	Invitrogen
BL21λDE3	F^−^ *omp*T *gal* (*dcm*) (*lon*) *hsd*S_B_(r_B_^−^ m_B_^−^) *endA1 hsdR1*7(r_K_^−^m_K_^+^)	Invitrogen
*S. suis* serotype 2		
P1/7	Virulent strain isolated from a pig with meningitis	[58]
31533	Virulent strain isolated from a pig with meningitis	[10]
166′	Virulent strain isolated from a pig with meningitis	[59]
D282	Virulent strain isolated from a diseased pig	[60]
S735	Serotype 2 reference strain, isolated from a pig with pneumonia	[61]
24	Virulent strain isolated from a human case of meningitis	[62]
LEF95	Virulent strain isolated from a human case of meningitis	[63]
HUD Limoges	Virulent strain isolated from a human case of septic-shock	[64]
89–1591	Virulent field strain isolated from a pig with septicemia	[65]
Δ*sipF*	Derived from P1/7. In frame deletion of *sipF*	This work
Δ*sfp2*	Derived from P1/7. In frame deletion of *sfp2*	This work
Δ*sfp1*	Derived from P1/7. In frame deletion of *sfp1*	This work
Δ*srtF*	Derived from P1/7. In frame deletion of *srtF*	This work
Δ*srtA*	Derived from P1/7. Deletion of *srtA*	This work
Δ*srtA*comp*srtA*	Δ*srtA* complemented with pSAcomp1	This work
Δ*srtA*compmock	Δ*srtA* transformed with empty pSET-3	This work
Plasmids		
pCR2.1	Ap^r^, Km^r^, *oriR*(f1) MCS *oriR* (ColE1)	Invitrogen
pDIA17	Cm^r^, *oriR* pACYC184, Tet promoter Δ*lacI*	[66]
pIVEX2.4d	Ap^r^, *oriR* pUC, T7 promoter, His-Tag coding sequence	Roche Applied Science
pSET4s	Thermosensitive vector for allelic replacement is *S. suis*. Replication functions of pG+host3, MCS *oriR* pUC19 *lacZ* Sp^R^	[49]
p4Δ*sipF*	pSET4s carrying the construct for *sipF* allelic replacement	This work
p4Δ*sfp2*	pSET4s carrying the construct for *sfp2* allelic replacement	This work
p4Δ*sfp1*	pSET4s carrying the construct for *sfp1* allelic replacement	This work
p4Δ*srtF*	pSET4s carrying the construct for *srtF* allelic replacement	This work
pSAD1	pSET4s carrying *cat* flanked by 5′ and 3′ ends of *srtA*	[43]
pSAD11	pSAD1 devoid of *cat*	This work
pSET-3	*S. suis-E. coli* shuttle vector	[50]
pSAcomp1	pSET-3 carrying the full length *srtA* gene under the control of *cat* promoter	[43]

As mentioned above, Sfp2 could neither be detected in extracts of whole cell or cell wall proteins, nor in concentrated culture supernatant fractions of the additional strains under investigation. Consistently, when we sequenced the *srtF* cluster in these strains, the same ochre nonsense mutation found in strain P1/7 was detected in the *sfp2* gene (GenBank Accession numbers GQ279101 to GQ279107).

### The *srtF* Pilus is Dispensable for Adhesion to and Invasion of Porcine BMEC

**Figure 7:**
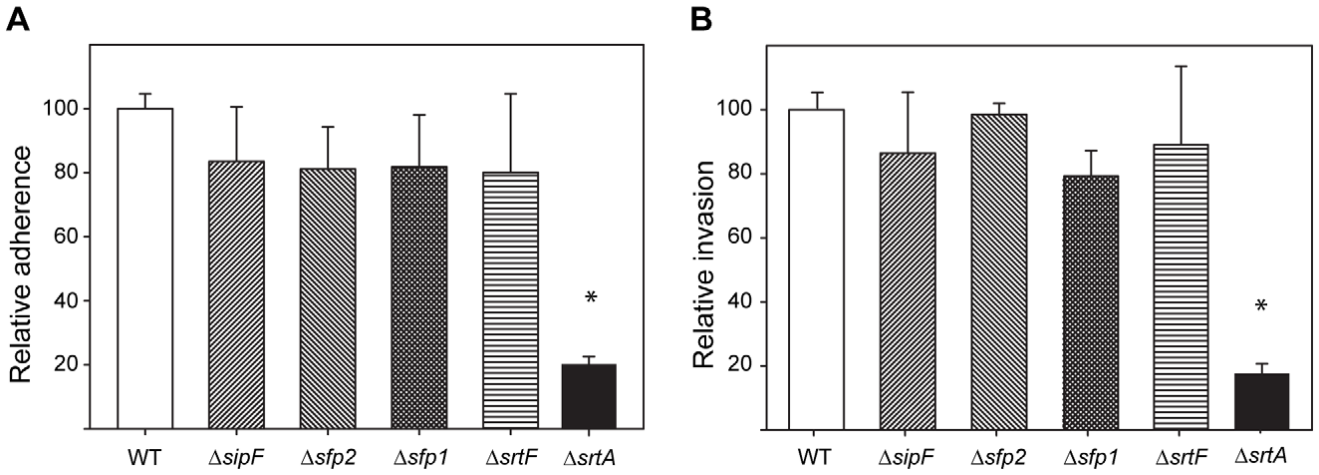
Interactions of the WT piliated strain P1/7 and derived mutants with porcine BMEC. The mutants showed similar levels of adherence to (A) and invasion of (B) porcine BMEC. Data for the WT strain (adherence = 1.59×10^5^±1.42×10^4^ recovered CFU/well; invasion = 1.79×10^3^±1.54×10^2^ recovered CFU/well) have been normalized to 100%. Data are from at least 4 independent experiments. Error bars show the standard error of the mean. No significant differences were found between the WT and the *srtF* cluster mutant strains, while the Δ*srtA* mutant, used as an internal control, showed significant differences in both adherence and invasion (t-test, P<0.05, indicated by the asterisk).

The first gene of the *srtF* pilus cluster, *sipF*, has been found to be highly upregulated by *S. suis* upon contact with cultured porcine BMEC, which are a major type of cells forming the BBB [13]. GBS pili have been shown to be important for adhesion of that pathogen to human BMEC, although in GBS adhesion required the presence of the ancillary subunit [28]. Despite the fact that the homologous putative adhesin Sfp2 is missing from *S. suis* pili encoded by the *srtF* cluster, we investigated the contribution of these pili to the adherence to and invasion of porcine BMEC using in vitro assays. As expected for an adhesin-less pilus, Figure 7A shows that there were no significant differences between the WT and mutant strains regarding *S. suis* adherence to porcine BMEC. A similar absence of differences between the WT and mutant strains was observed when invasion of porcine BMEC by the WT and mutant strains was evaluated (Figure 7B). In contrast, a Δ*srtA* mutant was severely impaired in its interactions with porcine BMEC, as previously reported [30].

### Abolishment of Pili Production Does Not Impair *S. suis* Sepsis in the Mouse

**Figure 8:**
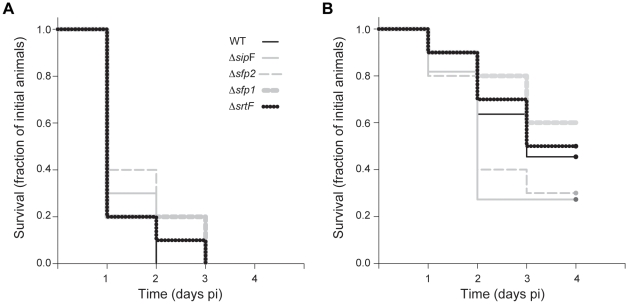
Survival of mice inoculated with the WT piliated strain P1/7 and derived mutant strains. No significant differences (LogRank test; p>0.3) in survival were observed between groups at (A) high (5×10^7^ CFU/animal) or (B) intermediate (1×10^7^ CFU/animal) doses of inoculation.

Using a GBS mutant that produces adhesin-less pili, it has been shown that the pilus backbone itself promotes phagocyte resistance and systemic virulence [25]. On the basis of these data, we hypothesized that the adhesin-less *srtF* pilus cluster described here might contribute to *S. suis* sepsis. To assess this hypothesis, we performed in vivo trials using a validated CD1 mouse model of *S. suis* infection that uses the intraperitoneal route of inoculation [11]. In a first experiment, mice received 5×10^7^ CFU of the WT or mutant strains. Most mice in the WT and the mutant groups presented severe clinical signs associated with septicemia, such as depression, swollen eyes, weakness and prostration during the first 24 h post-inoculation (pi). Most mice died from septicemia in all groups during the first 2 days of the trial and the remaining animals were killed for ethical reasons at day 3 pi (Figure 8A). Moreover, *S. suis* could be isolated at high titers (>1×10^7^ CFU/ml) from blood samples and organs such as the liver and spleen of septicemic animals in all the groups (>1×10^7^ CFU/0.5 g of tissue in some animals) (data not shown). These results suggest that pili encoded by the *srtF* pilus cluster are not major mediators of *S. suis* sepsis. However, the high dose of inoculation used may not allow for discerning more modest contributions of the pilus structures to the virulence of this pathogen. To address this concern, we performed a second in vivo trial using a lower dose of inoculation (1×10^7^ CFU). At this lower dose, animals in the WT and the mutant groups showed, overall, less severe clinical signs than in the previous experiment conducted with the higher dose. Moreover, death was delayed and strongly reduced and several animals in all the groups survived the trial. However, as in the previous trial, no significant differences in the severity of the clinical signs, bacterial isolation from blood and organs (data not shown), nor in mortality (LogRank test, p = 0.388) (Figure 8B), were observed between the WT and mutant groups. Taken together, the in vivo trial results strongly argue that pili encoded by the *srtF* cluster may be dispensable for *S. suis* sepsis.

## Discussion

The presence of thin pilus-like structures on the surface of *S. suis* was noticed as early as 1990 by ultrastructural studies using electron microscopy [30]. However, only very recently Fittipaldi et al. identified a first pilus cluster in *S. suis*, later renamed by Takamatsu et al. as the *srtF* pilus cluster [13], [18]. Results presented here show that in the highly virulent *S. suis* strain P1/7 and in other well characterized virulent serotype 2 strains the *srtF* pilus cluster encode pili which are formed by the major pilin subunit Sfp1 only. Indeed, Western-blotting of *S. suis* cell wall proteins with antibodies directed against the Sfp1 subunit clearly showed, for these WT strains, the presence of polymers of the Sfp1 pilin subunit, compatible with a pilus structure in which Sfp1 constitutes the backbone. Moreover, IEM results for strain P 1/7 showed that the gold particles were localized on the *S. suis* surface in pilus-like appendages. Consistently, these structures were absent from a Δ*sfp1* mutant.

It has been shown that in Gram-positive bacteria polymerization of pili is driven by specific, dedicated sortases encoded by genes that are clustered together with the genes encoding the pilin subunits they polymerize, while attachment to the cell wall peptidoglycan is mediated by the housekeeping sortase [16], [31]. In agreement, our results demonstrate that SrtF drives the polymerization of the pilus encoded by the *S. suis srtF* cluster. In fact, Sfp1 monomers, but not Sfp1 polymers, were detected on the cell wall of the corresponding Δ*srtF* mutant. These monomers are likely to be attached to the peptidoglycan through the action of SrtA, as suggested by the fact that they are absent from the cell wall fraction but more abundant in the culture supernatant of the corresponding Δ*srtA* mutant. In this latter mutant, in agreement with the proposed transient, non-covalent anchoring of pili to the cell wall by the dedicated sortases in a housekeeping sortase-less genomic background [16], [27], [29], [32], polymerized pili could be observed in the cell wall fraction. However, Sfp1 polymers were released in higher amounts to the culture supernatant by the Δ*srtA* mutant in comparison to the WT strain. Moreover, pili produced by the Δ*srtA* mutant were, in average, much longer than those observed in the WT parent strain. This observation might imply a role for the housekeeping sortase SrtA in termination of the chain. Additional experiments are needed to ascertain this hypothesis.

To our knowledge, the role in pili biogenesis of signal peptidase-like enzymes found in pilus clusters has only been investigated for the GAS T3 and the *S. pneumoniae* PI-2 pili [22], [33], [34]. It was observed that these signal peptidases (named SipA in the former two species) were essential for pili polymerization and assembly [22], [33], [34]. However, inactivation of the *S. suis sipF* gene did not prevent the polymerization of the pilin backbone subunit Sfp1. It has been suggested that GAS SipA is unlikely to function as a signal peptidase, since it lacks the highly conserved and catalytically important serine and lysine residues of these enzymes [33]. Instead, a putative chaperone function for GAS SipA has been proposed [33]. In contrast, *S. suis* SipF does possess these two conserved residues (data not shown). Therefore, it might be hypothesized that *S. suis* SipF may function as a signal peptidase involved in the removal of the Sfp1 signal peptide leader. However, since the export of Sfp1 was not prevented by deletion of *sipF*, the possibility of other enzymes with signal peptidase activity (most likely the *S. suis* housekeeping signal peptidase) compensating the function of SipF should be envisaged. This hypothetic alternative processing is, however, likely to hamper the efficient polymerization of the pilin subunits, as shown by the fact that the intensity of the higher molecular weight Sfp1-polymers was lower in the Δ*sipF* mutant than it was in the WT strain (Figure 2). Studies are currently being carried out in our laboratories to further understand the participation of SipF in the polymerization of Sfp1.

In this study, with the exception of the Δ*srtA* mutant, we have not complemented the mutant strains with the WT genes that each is missing. However, since the mutants were generated by precise, in frame deletions that were verified by sequencing, we consider very unlikely the possibility of polar effects. In fact, inactivation of *sipF* and *sfp2* (the more upstream genes of the *srtF* cluster) did not result in phenotypic changes, while eventual polar effects in the Δ*sfp1* mutant affecting also *srtF* expression, would have lead to a similar phenotype than that caused by disruption of *sfp1* alone. Further, our results support new genome sequence data indicating that *S. suis* P1/7 *sfp2* is a pseudogene [14], [18]. Importantly, this genetic organization with a non functional *sfp2* gene is shared by strains of *S. suis* serotype 2 belonging to the more virulent multilocus sequence typing ST1 complex [35], such as the recently sequenced Chinese isolates 98HAH12, 05ZYH33 and GZ1 [36], [37]. In fact, in silico analysis suggests that *sfp2* is also a pseudogene in these strains (data not shown). Furthermore, when we sequenced other well-characterized virulent *S. suis* serotype 2 strains (many of them belonging to the same ST1 complex), they presented the same ochre nonsense mutation in the *sfp2* gene. Consistently, these strains did not produce the Sfp2 pilin subunit. Nevertheless, the possibility of some *S. suis* strains expressing pili formed by both Sfp1 and Sfp2 subunits cannot be excluded. In fact, it has recently been reported that a Spanish *S. suis* isolate may display Sfp2 on its surface [38]. However, it is worth noting that Sfp2 was identified in that Spanish isolate by shaving the surface of bacteria with proteases followed by LC/MS/MS analysis of the resulting peptides and homology comparisons, and not by the use of mutagenesis and specific antibodies directed against that protein [38]. Moreover, that study surprisingly suggested that Sfp2 might form the backbone of the pilus encoded by the *srtF* cluster of that isolate [38]. However, that possibility is not supported by homology comparisons to the pili clusters of other Gram-positive bacteria [17], [22], [23], [24], nor is by data presented in the present study, which clearly show that Sfp1 constitutes the backbone of pili encoded by the *srtF* cluster.

We report here that pili encoded by the *srtF* pilus cluster of *S. suis* strain P1/7 are factors dispensable for adherence to porcine BMEC. They also are of minor importance for invasion of these cells. These results were not unexpected, since the *srtF* pilus lacks the pilin subunit Sfp2, which is, on the basis of homology comparisons, the putative pilin adhesin. To date, all described Gram-positive pili have at least one functional ancillary protein, and several groups of investigators have demonstrated that ancillary proteins play a major role in adhesion to host cells [22], [28], [31], [39]. Indeed, deletion of the putative pilin adhesin in GBS resulted in a mutant strain expressing pili composed of the major subunit only, which showed impaired interactions with human BMEC [28]. Moreover, other studies using streptococcal mutants reported that pili formed only by the major pilin subunit had diminished adhesive capacities compared to their respective parental strains [31], [39]. In this regard, and despite our results for strain P1/7 and other strains analyzed in this study, it should be noted that we cannot rule out the possibility that the *srtF* cluster would contribute to adherence to and/or invasion of porcine BMEC if the putative adhesin Sfp2 were expressed, as it might plausibly be the case in other *S. suis* strains. In addition, although it is an unlikely hypothesis, since we have only tested interactions with porcine BMEC, we cannot exclude that pili formed only by Sfp1 might play a role in adhesion to other cell types.

The murine model used in this study has proven reliable and reproducible and constitutes an excellent alternative to the use of porcine models of infection [7], [8], [11], [40], [41], [42]. However, this model uses the intraperitoneal route of inoculation [11] and, therefore, it overlooks the initial colonization of the upper respiratory tract by *S. suis*. Consequently, results of the murine trials presented here should be interpreted within these limitations. However, from our in vitro and in vivo results it may be advanced that pili encoded by the *srtF* cluster may not be critical for the full virulence of *S. suis* strain P 1/7. Indeed, the nonpiliated Δ*sfp1* mutant as well as the Δ*srtF* mutant (which expresses Sfp1 monomers but not polymers in its surface) induced as strong sepsis in the mouse as did the WT strain. In addition, since no differences in the interactions with porcine BMEC were observed between the WT and the mutant strains, participation of pili in the first steps of *S. suis* meningitis might be unlikely. Interestingly, besides the *srtF* cluster, only two other putative pili clusters are found in strain P1/7 (Fig. S2) and none of them seem likely to be able to mediate pili formation [18]. Indeed, the first of these additional clusters, designated as the *srtE* cluster, comprises a putative signal peptidase (SSU_0450) as well as the *srtE* gene (SSU_0453). However, this cluster lacks genes encoding the major and the ancillary pilin subunits. Instead, similar to the reported organization of the cluster in the *S. suis* serotype 2 reference strain [43], in strain P1/7 a putative exported protein (SSU_0451) and a transposase fragment (SSU_0452) are found between *sipF* and *srtE* [18 and unpublished data]. The remaining pilus cluster of strain P1/7 is homologous to the *rlrA* pilus island of *S. pneumoniae* TIGR4 [41]. This cluster, named *srtBCD* cluster, contains three sortase-like genes (*srtB*, *srtC*, and *srtD*) and four other genes (designated *sbp1*, *sbp2*, *sbp3*, and *sbp4*), which putatively encode putative cell-wall anchor family proteins containing pilin motifs, E boxes and/or CWSSs [18]. However, *sbp2*, which encodes the putative backbone subunit, is truncated by a nonsense mutation in strain P1/7 [18], leading to the notion that pili cannot be expressed from this cluster. Importantly, further analysis of a large collection of serotype 2 strains indicated that all tested strains of this serotype possessed an incomplete *srtE* cluster similar to that found in strain P1/7, and that they presented the same or other nonsense mutations in the *sbp2* gene encoding the major subunit of the *srtBCD* cluster [18 and unpublished data]. Taken together with our in vivo evaluation in the mouse, these data suggest that pili in general might likely be dispensable for the full virulence of this highly virulent invasive isolate.

Several reports have shown that pili fulfill a myriad of virulence-related functions in different streptococcal pathogens [16], [25], [44]. However, the notion of pili being essential for streptococcal virulence has not been systematically evaluated from an epidemiological perspective. To our knowledge, only a few studies have so far analyzed the correlation between production of pili and virulence using a large number of isolates recovered from the field [22], [44], [45]. In a first study, the pilin-encoding gene *rrgC* (a member of the *rlrA* pilus cluster) was absent from 78% of 484 virulent *S. pneumoniae* strains tested [44]. In addition, the presence of *rrgC* per se did not appear to be associated with increased virulence, since, when present, the gene was found at similar frequencies in nasopharyngeal and septicemic isolates [44]. Similar results (*rlrA* pilus cluster present in only 27% of the virulent strains tested) were obtained when analyzing an essentially different pneumococcal collection of invasive isolates [45]. Finally, a second *S. pneumoniae* pilus (PI-2) was found to be of low prevalence (16%) among clinical isolates [22]. Taken together, these results suggest that despite published data obtained from mouse studies, pili might in fact not represent a central virulence factor for *S. pneumoniae* invasive disease in humans. These *S. pneumoniae* reports [22], [44], [45] sustain therefore findings presented in the present study suggesting that pili may be not critical for the full virulence of some highly invasive *S. suis* isolates. It is to expect that future epidemiological studies carried out with *S. suis* and other streptococci will shed light on the actual contribution of pili to the virulence traits of pathogenic members of this important genus.

## Materials and Methods

### Ethics Statement

All animals used in this study were treated, and trials conducted, in accordance with the guidelines and policies of the Canadian Council on Animal Care (CCAC), enforced locally by the Ethics Committee of the Faculté de médecine vétérinaire of the Université de Montréal. The protocols and procedures were approved by the Ethics Committee.

### Bacterial Strains, Plasmids, Media, Culture Conditions and Reagents

Bacterial strains and plasmids used in this study are listed in Table 1. Unless otherwise indicated, *S. suis* strains were grown in Todd-Hewitt (Becton Dickinson, Sparks, MD) broth (THB) or agar (THA) at 37°C. *E. coli* strains were grown in Luria-Bertani (LB) broth or agar (Becton Dickinson) at 37°C. When needed, antibiotics (Sigma, Oakville, ON, Canada) were added to the culture media at the following concentrations: for *S. suis*: spectinomycin (Sp) at 100 µg/ml; for *E. coli*: kanamycin (Km) and Sp at 50 µg/ml; chloramphenicol (Cm) at 30 µg/ml and ampicillin at 100 µg/ml. Unless otherwise indicated, all reagents used in this study were purchased from Sigma.

### DNA Manipulations

*S. suis* genomic DNA was prepared by the guanidium thiocyanathe method [46]. Minipreparations of recombinant plasmids and transformation of *E. coli* were performed by standard procedures [47]. Restriction enzymes and DNA-modifying enzymes were purchased from TaKaRa Bio (Otsu, Shiga, Japan) and used according to the manufacturers' directions. PCR reactions were carried out with the iProof proofreading DNA polymerase (BioRad Laboratories, Hercules, CA) or with Taq DNA polymerase (GE Healthcare, Piscataway, NJ). Oligonucleotide primers were from Invitrogen (Burlington, ON, Canada). Amplification products were purified on Sephadex S-400 columns (GE Healthcare) and sequenced with an ABI 310 automated DNA sequencer, using the ABI PRISM dye terminator cycle sequencing kit (Applied Biosystems, Foster City, CA).

### Construction of Deletion Mutants

Precise, in-frame deletions in *sipF*, *sfp2*, *sfp1 and srtF* were constructed by using splicing-by-overlap-extension PCR [48]. The primers used for the construction of deletion alleles are listed in Table S1. Appropriate deletion alleles generated by PCR were cloned into plasmid pCR2.1 (Invitrogen), extracted with BamHI and PstI and recloned into the thermosensitive *E. coli-S. suis* shuttle vector pSET4s [49] digested with the same enzymes. Plasmid pSAD11 used in this study for deletion of *srtA* was obtained by EcoT22I removal of the *cat* gene from plasmid pSAD1, previously used for insertional inactivation of *srtA* [43]. Complementation of the Δ*srtA* mutant was achieved by electroporation of the mutant with the previously described pSAcomp1 vector [43]. This mutant was also mock-complemented with empty pSET-3 vector from which pSAcomp1 is derived [43], [50]. Electroporation of *S. suis* and procedures for isolation of mutants were those described previously [50]. Deletions of all genes were confirmed by PCR and sequencing analysis.

### Expression and Purification of Recombinant 6xHis-Sfp1 and 6xHis-Sfp2 and Antibody Production

DNA fragments intragenic to *sfp1* and *sfp2* were generated by PCR using genomic DNA of *S. suis* strain P1/7 as template and the primer pairs Sfp1-fwd/Sfp1-rev (for *sfp1*) and Sfp2-fwd/Sfp2-rev and Sfp2-fwd2/Sfp2-rev2 (for *sfp2*), respectively (Table S1 and Figure 1). PCR amplicons were digested with NdeI and BamHI and cloned into plasmid pIVEX 2.4d (Roche Applied Science, Laval, QC, Canada), digested with the same enzymes. The resulting recombinant plasmids were introduced into *E. coli* TOP 10 (Invitrogen) for sequence analysis and storage and into *E. coli* BL21λDE3/pDIA17 for protein expression. Induction was carried out with IPTG, as previously described [51]. Recombinant 6xHis-proteins were purified under denaturing conditions by affinity chromatography on Ni-NTA columns (Protino protein purification system, Macherey-Nagel, Düren, Germany) according to the manufacturers' recommendations. Protein purity was checked on SDS-PAGE and accurate protein concentrations were determined by a simplified Lowry test [52]. Rabbit polyclonal antibodies (pAb) against the individual proteins were produced as previously described [53]. The specificity of each antibody was determined by Western blotting against the purified 6xHis-proteins as well as against crude *S. suis* cell extracts prepared from the WT and mutant strains.

### Cell Wall, Whole Cell and Culture Supernatant Protein Preparations

*S. suis* strains were grown in 10 ml of THB at 37°C. Bacteria were harvested by centrifugation during the late exponential phase of culture and resuspended in 220 µl spheroplasting buffer [10 ml spheroplasting buffer: 24 mg Tris, 20 mg MgCl_2_6H_2_O, 2.6 g raffinose, 5000 U mutanolysin, one capsule Complete Mini EDTA-free protease inhibitor cocktail (Roche)], as described [54]. The digestion was performed for 1 h at 37°C under gentle agitation. After centrifugation at 13,000×g for 15 min at 4°C, supernatants corresponding to the cell wall fractions were analyzed on SDS-PAGE. Total proteins extracts were prepared as previously described [55]. Supernatants obtained after centrifugation of ON *S. suis* cultures grown in THB were concentrated 10-fold by Ultrafree-MC centrifugal filter devices (Millipore Corp., Bedford MA, USA).

### Immunogold Electron Microscopy

*S. suis* WT and mutant strains were grown overnight at 37°C in 10 ml of THB, harvested by centrifugation, and resuspended in 250 µl of 1% glutaraldehyde in phosphate-buffered saline (PBS), pH 7.3. After fixation for 30 min, 20 µl of the bacterial suspensions were placed on nickel-Formvar grids (Canemco, Lakefield, QC, Canada) and allowed to partially air dry. Grids were subsequently blocked for 30 min with 10% normal donkey serum (Jackson ImmunoResearch) in dilution buffer (PBS containing 1% bovine serum albumin and 1% Tween-20, pH 7.3). Thereafter, samples were soaked in 50 µl of anti-Sfp1 or anti-Sfp2 specific pAb or control rabbit normal serum diluted 1/10 in dilution buffer for 2 h. The grids were then washed 5 times with dilution buffer, soaked in 50 µl of 10 nm colloidal gold-goat anti-rabbit IgG (Sigma) diluted 1/20 in dilution buffer, and incubated for 1 h. After three washes with PBS, grids were stained with 2% uranyl acetate for 30 s, and observed with a JEM-1230 electron microscope (JEOL Ltd, Tokyo, Japan) at an accelerating voltage of 80 kV.

### Adherence to and Invasion of Porcine BMEC

The porcine BMEC cell line PBMEC/C1-2 [56] was grown in Primaria 24-well tissue culture plates (Becton Dickinson, Franklin Lakes, NJ) using IF culture medium (a mixture of 1∶1 Iscove's modified Dulbecco's and Ham's F-12 media, Invitrogen) supplemented as previously described [10]. *S. suis* strains were grown in THB for 16 h at 37°C, harvested by centrifugation, washed twice in PBS, and resuspended in fresh IF culture medium. The invasion assays were performed as described previously [10]. Briefly, confluent monolayers of porcine BMEC at 10^5^ cells/well were infected with 1-ml aliquots of bacterial suspensions at 10^5^ CFU/ml (multiplicity of infection of 1). The plates were centrifuged at 800×g for 10 min and incubated for 2 h at 37°C under 5% CO_2_. The monolayers were then washed twice with PBS, 1 ml of cell culture medium containing 100 µg/ml of gentamicin and 5 µg/ml of penicillin G was added to each well, and incubation continued for 1 h. After incubation, monolayers were washed three times with PBS, trypsinized and disrupted by repeated pipetting. Serial dilutions of the cell lysates were plated onto THA and incubated overnight at 37°C. Invasion rates were calculated as the number of bacteria remaining after the antibiotic treatment with respect to the total number of inoculated bacteria. Adherence assays were performed essentially as described for invasion, but no antibiotic treatment was performed. After incubation for 2 h, cells were washed five times with PBS, trypsinized, disrupted, and serial dilutions of the cell lysates were plated as described above. Adherence rates were calculated as the number of bacteria remaining attached to cells after the incubation period with respect to the total number of inoculated bacteria.

### Experimental Infection of Mice

A validated CD1 murine model of *S. suis* infection was used [11]. In a first experiment, 50 female, 6-week old CD1 mice (Charles River Laboratories, Wilmington, MA) were divided in 5 groups of 10 animals (day 0). Group 1 was inoculated by intraperitoneal injection of 1 ml of *S. suis* strain P1/7 suspension at 5×10^7^ CFU/ml, while groups 2, 3, 4 and 5 received the same dose of mutant strains Δ*sipF*, Δ*sfp2*, Δ*sfp1* and Δ*srtF*, respectively. Mice were monitored 3 times/day for 3 days for clinical signs and assigned clinical scores as previously described [11]. Blood samples (5 µl) were collected daily from the tail vein and at necropsy by cardiac puncture and used to evaluate bacterial load by plating onto sheep blood agar plates. Isolated tiny α-hemolytic colonies were counted and assigned to *S. suis* by serotyping as previously described [57]. At necropsy, macroscopic examination was performed. Bacterial colonization of the liver, spleen and brain of infected animals was also evaluated. Briefly, small pieces of these organs weighing 0.5 g were trimmed, placed in 500 µl of PBS and homogenized. Thereafter, 50 µl of the suspensions were plated as described above. A second experiment was carried out essentially as described above but the mice received a lower dose (1 ml of 1×10^7^ CFU/ml) of *S. suis* P1/7 or mutant strains. In this second experiment, the WT and Δ*sipF* groups comprised 11 mice each while the other 3 groups (Δ*sfp2*, Δ*sfp1* and Δ*srtF*) were of 10 mice each. Animals were monitored as described above for 4 days.

## Supporting Information

Figure S1 PCR amplification of the *srtF* cluster in different *S. suis* serotype 2 strains using specific primers annealing upstream of *sipF* and downstream of *srtF*. All strains were positive for an 8.34 kb fragment, with the exception of North American strain 89–1591, which had been found not to produce Sfp1 monomers. Lane 1: Strain P1/7. Lane 2: Strain 31533. Lane 3: Strain 166. Lane 4: Strain D24. Lane 5: Strain S735 (serotype 2 reference strain). Lane 6: Strain D282. Lane 7: Strain LEF95. Lane 8: Strain HUD Limoges. Lane 9: Strain 89–1591. Lane 10, no DNA template.
(0.57 MB PDF)

Figure S2 Pilus clusters found in the genome of strain.
(0.30 MB PDF)

Table S1 (0.05 MB DOC)
