# Supplementary figures and images for: Mutations in the Gene Encoding the Ancillary Pilin Subunit of the Streptococcus suis srtF Cluster Result in Pili Formed by the Major Subunit Only
Source: PLoS One. 2010 Jan 5;5(1):e8426. doi: 10.1371/journal.pone.0008426 (PMC2797073; doi:10.1371/journal.pone.0008426)

1

2

3

4

5

6

7

8

9

10

9.34 kb ➔

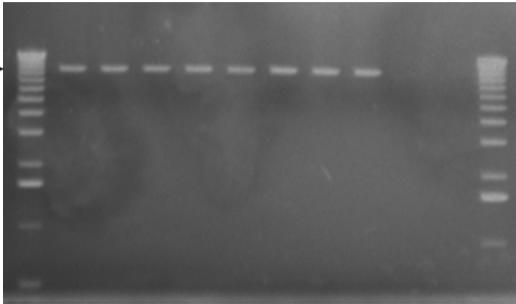

Supplement: Figure S1 — PCR amplification of the srtF cluster in different S. suis serotype 2 strains using specific primers annealing upstream of sipF and downstream of srtF. All strains were positive for an 8.34 kb fragment, with the exception of North American strain 89–1591, which had been found not to produce Sfp1 monomers. Lane 1: Strain P1/7. Lane 2: Strain 31533. Lane 3: Strain 166. Lane 4: Strain D24. Lane 5: Strain S735 (serotype 2 reference strain). Lane 6: Strain D282. Lane 7: Strain LEF95. Lane 8: Strain HUD Limoges. Lane 9: Strain 89–1591. Lane 10, no DNA template. (0.57 MB PDF) [file pone.0008426.s001.pdf]
