# Supplementary material for: Mutations in the Gene Encoding the Ancillary Pilin Subunit of the Streptococcus suis srtF Cluster Result in Pili Formed by the Major Subunit Only
Source: PLoS One. 2010 Jan 5;5(1):e8426. doi: 10.1371/journal.pone.0008426 (PMC2797073; doi:10.1371/journal.pone.0008426)
Supplement: Figure S2 — Pilus clusters found in the genome of strain. (0.30 MB PDF) [file pone.0008426.s002.pdf]

3 pilus clusters are present in *S. suis* strain P1/7

srtBCD pilus cluster

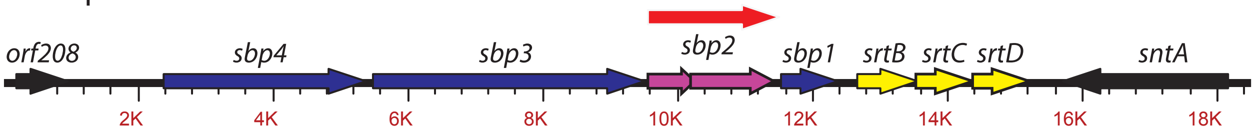

In strain P1/7, *sbp2*, encoding the main pilin subunit, is interrupted by a nonsense mutation resulting in two ORFs (depicted in pink), which are predicted to be non-functional (Takamatsu et al. Vet Microbiol, 2009. 138:132-9)

srtE pilus cluster

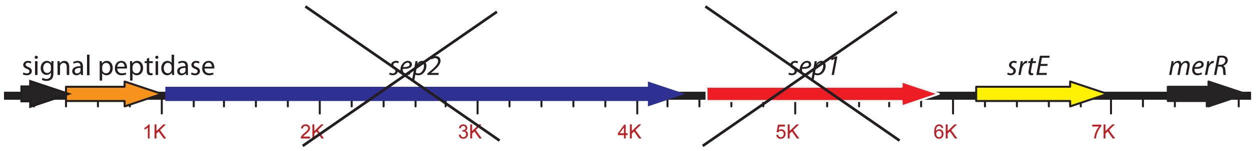

Depicted here is the *srtE* pilus cluster of *S. suis* strain DAT 294. In strain P1/7, both *sep1* and *sep2* are missing, suggesting a non-functional cluster (Takamatsu et al. Vet Microbiol, 2009. 138:132-9)

srtF pilus cluster

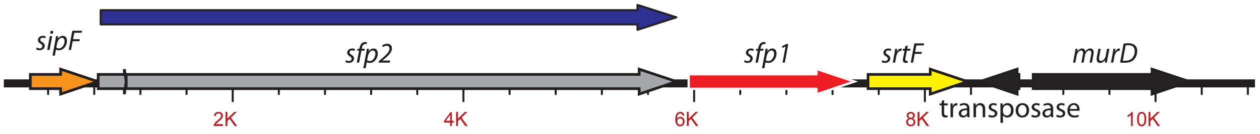

The third pilus cluster of *S. suis* is the *srtF* pilus cluster, which is characterized in the present study.

■ Ancillary subunit ■ Main subunit ■ Sortase ■ Signal peptidase
