# Supplementary material for: Mutations in the Gene Encoding the Ancillary Pilin Subunit of the Streptococcus suis srtF Cluster Result in Pili Formed by the Major Subunit Only
Source: PLoS One. 2010 Jan 5;5(1):e8426. doi: 10.1371/journal.pone.0008426 (PMC2797073; doi:10.1371/journal.pone.0008426)
Supplement: Table S1 — (0.05 MB DOC) [file pone.0008426.s003.doc]

Table S1. Oligonucleotide primers used in this study.

| Primer name | Sequence (5’ – 3’) | Use |
| --- | --- | --- |
| Inter-ID1 | CTGGGGATTGGGTGGATAT | Mutant construction |
| Inter-ID2 | TCTTGGTCGGTCTGGGGTTT | Mutant construction |
| Inter-ID3 | CAGAAAACAGACCTGGCCTG | Mutant construction |
| Inter-ID4 | GAACCAAGTATCACCACCAA | Mutant construction |
| Inter-ID5 | TTTGTCATGGGAGATAACCG | Mutant construction |
| Inter-ID6 | TCCATCTCCGCCTGTCGCAGGAGATTGAAC | Mutant construction |
| Inter-ID7 | GTTCAATCTCCTGCGACAGGCGGAGATGGA | Mutant construction |
| Inter-ID8 | ACCACCAAAGTTCGTTGCAG | Mutant construction |
| SrtF-ID1 | GACAGGTGCAGAGTTCAAC | Mutant construction |
| SrtF-ID2 | GTAGGCTTCAATGTCGGATG | Mutant construction |
| SrtF-ID3 | CATAGGCTATTAGTTCGTGG | Mutant construction |
| SrtF-ID4 | TTTTCAAGCCAGAGGACAGA | Mutant construction |
| SrtF-ID5 | CTATGATGCAGCCAACAATG | Mutant construction |
| SrtF-ID6 | ATCATCCAACACTTGGAGCCATTTGATCAT | Mutant construction |
| SrtF-ID7 | ATGATCAAATGGCTCCAAGTGTTGGATGAT | Mutant construction |
| SrtF-ID8 | TCTGCCCGTAGCGCTAGTTC | Mutant construction |
| Sbp-ID1 | AGAATTGGCAGCACCTGATG | Mutant construction |
| Sbp-ID2 | TCCAATCGCACCAGTCAGTACTGCCACAAG | Mutant construction |
| Sbp-ID3 | CTTGTGGCAGTACTGACTGGTGCGATTGGA | Mutant construction |
| Sbp-ID4 | CGGATGCAGTCACATTCCAG | Mutant construction |
| PSF-ID1 | AATTAACTCTGATACATCGCCG | Mutant construction |
| PSF-ID2 | GTATTGCAAATGCTGCAACTAC | Mutant construction |
| PSF-ID3 | TCTCTATAATTGACGGGAGTGG | Mutant construction |
| PSF-ID4 | GACTCTTTCCGGTTATCTCCCATCCTTTCCCTTGAGATTTGAAC | Mutant construction |
| PSF-ID5 | GTTCAAATCTCAAGGGAAAGGATGGGAGATAACCGGAAAGAGTC | Mutant construction |
| PSF-ID6 | TTGACACTTGCTCAGCAGGG | Mutant construction |
| Sfp1-fwd | GTGGAGCAGGC**CATATG**ACTGTCTCT ^1^ | Cloning for protein expression |
| Sfp1-rev | CACTCCGTCA**GGATCC**TTGACAACCT ^2^ | Cloning for protein expression |
| Sfp2-fwd | CCTGCTGAG**CATATG**TCAACAGAGTC ^1^ | Cloning for protein expression |
| Sfp2-rev | GGTTGGCGGAGG**GGATCC**AATATTTGA ^2^ | Cloning for protein expression |
| Sfp2-fwd2 | GGAACTCAAGGA**CATATG**TATCGTTTCT ^1^ | Cloning for protein expression |
| Sfp2-rev 2 | TTGCTAGC**GGATCC**AGATGTTATGG ^2^ | Cloning for protein expression |

^1^ NdeI restriction site is in bold.

^2^ BamHI restriction site is in bold.
